# Fatal Infantile Cardiomyopathy Associated with a Homozygous *MYL2* c.413T>A (p.Met138Lys) Variant: A Case Expanding the Recessive *MYL2* Phenotypic Spectrum

**DOI:** 10.3390/genes17040441

**Published:** 2026-04-12

**Authors:** Mohammed Shahab Uddin, Yasmeen Alnamshan, Khaled Shafeen, Syeda Nilofer Jahan, Nora AlMadhi, Karthiga Gurumurthy, Abdullah Bin Hassan, Amr Esmail, Maryam AlQannas

**Affiliations:** 1 Department of Pediatric, Ministry of National Guard Health Affairs, Dammam 31412, Saudi Arabia; alnamshany2@mngha.med.sa (Y.A.); karthi.sangee86@gmail.com (K.G.); binhassanaa@mngha.med.sa (A.B.H.); alqannasma@mngha.med.sa (M.A.); 2 King Abdullah International Medical Research Center, Riyadh 11481, Saudi Arabia; 3 Faculty of Medicine, King Saud bin Abdulaziz University for Health Sciences, Riyadh 11426, Saudi Arabia; 4 Faculty of Medicine, Semmelweis University, 1094 Budapest, Hungary; khaledshafeen26@gmail.com; 5 Saveetha College of Nursing, Seveetha Institute of Medical and Technical Sciences, Chennai 602105, India

**Keywords:** cardiomyopathy, dilated, infant, myosin light chains, whole exome sequencing, mutation, missense, genotype–phenotype correlation

## Abstract

Background/Objectives: Infantile cardiomyopathy is a rare but often life-threatening condition in which monogenic causes are particularly relevant, especially when cardiac disease is preceded by hypotonia or multisystem involvement. Among sarcomeric genes, *MYL2*, encoding the ventricular regulatory myosin light chain, plays a critical role in myocardial contractility. However, biallelic *MYL2*-associated disease remains exceptionally rare, and its clinical spectrum is not fully defined. This study aims to describe a novel case and further delineate the phenotype of recessive *MYL2*-related cardiomyopathy. Methods: We report a male infant with congenital hypotonia and delayed motor development who underwent extensive metabolic, neuromuscular, and neuroimaging evaluation. Trio-based whole-exome sequencing was performed to identify a potential genetic etiology, followed by variant interpretation using standard bioinformatic and ACMG/AMP criteria. Results: The patient developed acute decompensated heart failure at approximately 10 months of age, with severe left ventricular systolic dysfunction and multiorgan failure, and died at 12 months despite maximal intensive care support. Whole-exome sequencing identified a homozygous *MYL2* c.413T>A (p.Met138Lys) missense variant. The variant is absent or extremely rare in population databases, affects a highly conserved residue, is predicted to be deleterious by multiple in silico tools, and is compatible with autosomal recessive inheritance, with both parents confirmed as heterozygous carriers. In the context of a phenotype consistent with recessive *MYL2*-associated disease, these findings support a likely pathogenic interpretation. Conclusions: This case expands the allelic and phenotypic spectrum of recessive *MYL2*-associated cardiomyopathy and highlights the value of early genomic testing in infants with unexplained hypotonia and rapidly progressive cardiac dysfunction. Molecular diagnosis may aid in prognosis, clinical decision-making, and genetic counseling.

## 1. Introduction

Infantile cardiomyopathy is an uncommon but highly morbid presentation in pediatric practice and remains an important cause of heart failure, intensive care admission, and early mortality. Although infectious, metabolic, and neuromuscular disorders may contribute to the phenotype, a substantial proportion of cases are now recognized to have a monogenic basis, particularly when cardiac disease occurs in association with hypotonia, developmental delay, or other multisystem features [1,2,3,4,5]. Accordingly, broader genomic testing has become increasingly relevant in infants with unexplained cardiomyopathy, where targeted panels may not fully capture the underlying genetic architecture [6,7,8,9]. Moreover, rapid sequencing in neonatal and pediatric critical care settings has shown meaningful diagnostic and clinical utility, including effects on management and counseling [10,11,12,13]. Genetic causes of infantile cardiomyopathy are heterogeneous and include variants in sarcomeric, cytoskeletal, desmosomal, mitochondrial, metabolic, and neuromuscular disease genes, as well as syndromic disorders. In early infancy, particularly when cardiomyopathy is accompanied by hypotonia, developmental delay, or multisystem involvement, broad genomic evaluation is often required because the differential diagnosis extends beyond classic isolated cardiomyopathy genes.

Within this context, *MYL2* encodes the ventricular regulatory myosin light chain, a key sarcomeric protein involved in stabilizing the myosin neck region and modulating cross-bridge kinetics, calcium sensitivity, and contractile performance [14,15]. Experimental studies have further shown that ventricular regulatory light-chain phosphorylation contributes to normal systolic function, whereas disruption of this pathway may impair force generation and promote ventricular dysfunction in model systems [16,17,18,19]. In parallel, biophysical analyses indicate that disease-associated *MYL2* variants can alter calcium binding, phosphorylation behavior, and strain-dependent mechanics, thereby providing a plausible link between sequence variation and cardiomyopathic phenotypes [20,21,22,23].

Clinically, heterozygous missense variants in *MYL2* have been described predominantly in autosomal dominant hypertrophic cardiomyopathy with variable penetrance [23]. By contrast, biallelic *MYL2* variants appear to define a much rarer and more severe recessive phenotype, often presenting in early life and sometimes accompanied by skeletal muscle involvement. A homozygous splice-site variant has been reported in association with congenital cardioskeletal myopathy and early cardiac death [24], and this allele was subsequently shown to result in reduced protein stability and abnormal sarcomeric incorporation [25]. Likewise, siblings carrying a homozygous C-terminal frameshift developed severe infantile cardiomyopathy and died early [26], whereas recessive *MYL2*-associated congenital fibre-type disproportion with cardiomyopathy has also been documented [27]. Collectively, these reports support an emerging recessive *MYL2* disease spectrum that is distinct from the more familiar dominant hypertrophic phenotype.

As genomic testing becomes increasingly integrated into acute pediatric care, careful case-based characterization remains essential for refining genotype–phenotype correlations in rare sarcomeric disorders. At the same time, interpretation of ultra-rare variants requires integration of population data, computational prediction, segregation, and phenotype specificity within established variant-classification frameworks [3,6,7,8,28,29]. Here, we report a fatal infantile cardiomyopathy associated with a homozygous *MYL2* c.413T>A (p.Met138Lys) variant, thereby expanding the allelic and phenotypic spectrum of recessive *MYL2*-related disease.

## 2. Case Report

**Figure 1:**
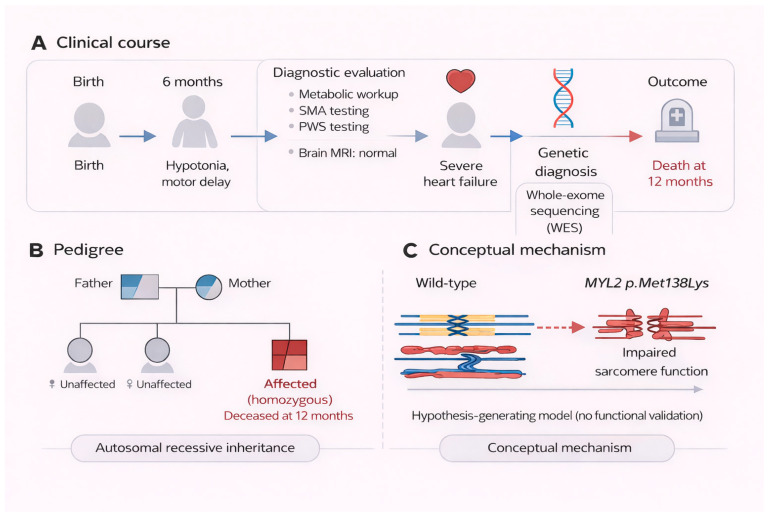
Clinical course, pedigree, and conceptual disease framework of the homozygous *MYL2* p.Met138Lys variant. (**A**) Clinical timeline of the proband from birth to death. Hypotonia and motor delay were recognized at approximately 6 months of age. Initial diagnostic evaluation, including metabolic screening, testing for spinal muscular atrophy (SMA) and Prader–Willi syndrome (PWS), and brain magnetic resonance imaging (MRI), was unrevealing. At approximately 10 months, the patient developed severe heart failure. Trio-based whole-exome sequencing subsequently identified a homozygous *MYL2* p.Met138Lys variant. The patient died at 12 months despite maximal supportive care. (**B**) Pedigree demonstrating segregation of the *MYL2* variant consistent with autosomal recessive inheritance. Both parents are heterozygous carriers, the affected child is homozygous, and the siblings are unaffected. (**C**) Conceptual schematic of the proposed disease mechanism. The *MYL2* p.Met138Lys variant may impair sarcomeric structure and contractile function, thereby contributing to cardiomyopathy. This model is hypothesis-generating and does not represent direct functional validation.

A male infant was brought to the emergency department in late infancy with 3 days of worsening lethargy, 1 week of poor oral intake, 2 days of vomiting, and progressive respiratory distress. On arrival, he appeared pale, tachypneic, and fatigued, with subcostal retractions and scattered rhonchi and crepitations on chest auscultation. Cardiac examination revealed a gallop rhythm. Electrocardiography demonstrated biatrial enlargement, left ventricular hypertrophy, and borderline QT prolongation. His weight and length were between the 10th and 25th percentiles, whereas head circumference was at the 50th percentile, without clear evidence of disproportionate growth failure. Echocardiography demonstrated markedly depressed left ventricular systolic function, with an ejection fraction of approximately 25%, left-sided chamber dilatation, pulmonary venous congestion, and mild mitral regurgitation without pericardial effusion. Although formal left ventricular z-scores, shortening fraction, right ventricular functional indices, and wall-thickness measurements were not available for this report, the overall echocardiographic picture was most consistent with severe dilated cardiomyopathy with advanced left ventricular failure at approximately 10 months of age. He was transferred to the pediatric intensive care unit, where he required endotracheal intubation, mechanical ventilation, inotropic support with milrinone and dobutamine, diuretics, broad-spectrum antibiotics, and close hemodynamic and fluid monitoring (Figure 1).

He was born at term by ventouse-assisted vaginal delivery after an uneventful pregnancy, with a birth weight of 2.9 kg and no reported perinatal complications. From early infancy, his parents noted generalized hypotonia, marked head lag, and delayed gross motor development. Between 6 and 9 months of age, he was unable to roll over or sit independently and had poor truncal tone, whereas social interaction and visual tracking were preserved for age. No dysmorphic features were recognized clinically. The parents were healthy, reportedly non-consanguineous, and had no known history of cardiomyopathy, heart failure, arrhythmia, sudden unexplained death, neuromuscular disease, or inherited metabolic disorder. His two older sisters were healthy and developmentally normal (Figure 1). The siblings were not genetically tested; therefore, their carrier status remains unknown. In view of the homozygous ultra-rare variant later identified, distant unrecognized relatedness or regional allele enrichment cannot be excluded.

Before the onset of overt cardiac failure, an extensive evaluation had been undertaken for the infant’s hypotonia and motor delay. Broad metabolic screening was unrevealing. Creatine kinase was within the normal range. Molecular testing for spinal muscular atrophy and methylation analysis for Prader–Willi syndrome was negative. Brain magnetic resonance imaging performed at 9 months of age demonstrated normal cerebral structure and myelination, without malformation or evidence of demyelination. A skeletal survey was also normal. Additional investigations for muscular dystrophy and primary myopathic disorders had been initiated but were still incomplete at the time of his acute deterioration. Thus, despite broad multidisciplinary assessment, no unifying diagnosis had been established, and the combination of hypotonia and delayed motor milestones remained unexplained (Figure 1).

Following admission, the child rapidly progressed to acute decompensated heart failure and required transfer to the pediatric intensive care unit. He underwent endotracheal intubation and mechanical ventilation and received inotropic support with milrinone and dobutamine, diuretic therapy, broad-spectrum antimicrobials, and close hemodynamic and fluid monitoring. Despite maximal medical therapy, his clinical course remained relentlessly progressive. Peak biochemical abnormalities during admission included lactate of 6.9 mmol/L, BNP or NT-proBNP approximately 25-fold above the upper reference range, and troponin approximately 30-fold above the upper reference range. He also developed rising liver enzymes and international normalized ratio, anemia with evidence of hemolysis, and progressive ventilator dependence, consistent with evolving multiorgan failure. Because of the severity of his cardiac dysfunction and the need for advanced pediatric cardiac intensive care, he was transferred to the National Guard Hospital in Riyadh. On arrival, he remained critically ill, intubated, and dependent on inotropic support, with persistent low cardiac output and acute liver failure.

Given the longstanding history of congenital hypotonia and gross motor delay, the absence of a metabolic or syndromic explanation, and the subsequent emergence of severe cardiomyopathy, a monogenic disorder was strongly suspected. Rapid trio whole-exome sequencing was therefore requested. Genomic DNA extracted from peripheral blood underwent exome enrichment targeting coding exons and flanking splice junctions of more than 20,000 protein-coding genes. Sequencing was performed on an Illumina platform with mean on-target coverage greater than 100×, and more than 98% of targeted bases were covered at ≥20× depth. Bioinformatic analysis included alignment to the GRCh37/hg19 reference genome, variant calling using a GATK-based pipeline, and annotation against population, disease, and in silico prediction databases. Variant prioritization focused on rare protein-altering alleles compatible with an autosomal-recessive model and the observed phenotype, with parental samples included for segregation analysis.

**Figure 2:**
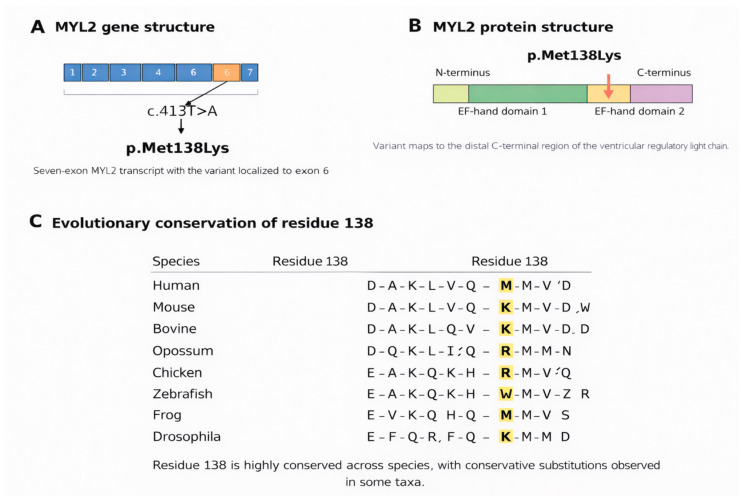
*MYL2* gene, protein structure, and evolutionary conservation of the p.Met138Lys variant. (**A**) Gene structure of *MYL2* showing the seven-exon organization. The identified variant c.413T>A is located in exon 6 and results in the missense substitution p.Met138Lys. (**B**) Schematic representation of the *MYL2* protein structure indicating the N-terminal and C-terminal regions and the EF-hand domains. The p.Met138Lys variant is positioned within the distal C-terminal region, corresponding to EF-hand domain 2. This localization supports potential disruption of calcium-binding or sarcomeric regulatory function. (**C**) Evolutionary conservation of residue 138 across species. A multiple-sequence alignment of representative orthologs demonstrates that residue 138 shows strong evolutionary conservation, with conservative substitutions observed in some taxa. The highlighted residues indicate the corresponding position across species, supporting the functional importance of this site.

**Figure 3:**
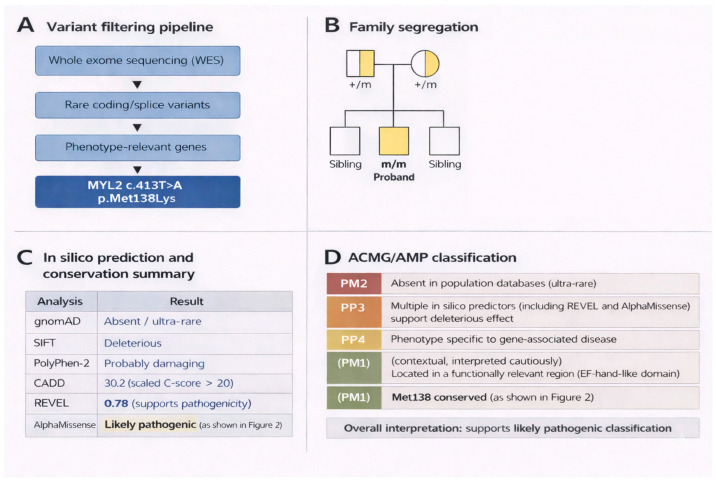
Variant prioritization, segregation, in silico prediction, and ACMG/AMP classification of *MYL2* p.Met138Lys. (**A**) Trio-based whole-exome sequencing workflow showing stepwise filtering from all variants to rare coding/splice-site variants and phenotype-relevant genes, identifying *MYL2* c.413T>A (p.Met138Lys). (**B**) Pedigree consistent with autosomal recessive inheritance: both parents are heterozygous carriers (+/m), and the proband is homozygous (m/m). (**C**) In silico prediction and conservation summary. The variant is absent or ultra-rare in population databases (gnomAD) and is predicted to be deleterious by SIFT, probably damaging by PolyPhen-2, to have a high CADD score, to support pathogenicity by REVEL, and to be likely pathogenic by AlphaMissense. Met138 is conserved across species (see Figure 2). (**D**) ACMG/AMP-oriented evidence summary. PM2, PP3, and PP4 support classification, while PM1 was considered cautiously based on localization within a functionally relevant EF-hand-like region and residue conservation. Overall, the findings support a likely pathogenic classification.

**Figure 4:**
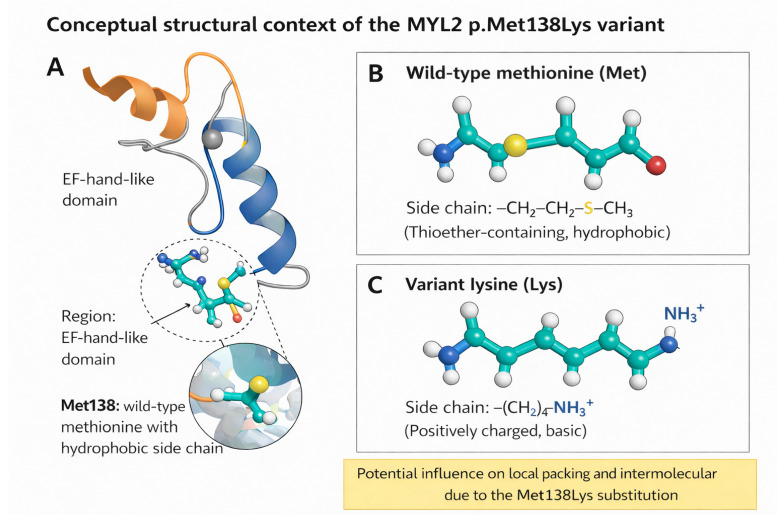
Conceptual structural context of the *MYL2* p.Met138Lys variant. (**A**) Schematic representation of the approximate location of Met138 within the C-terminal region of *MYL2*, shown in relation to EF-hand-like domain architecture. (**B**) Conceptual depiction of the wild-type methionine residue, highlighting its thioether-containing hydrophobic side chain and its possible contribution to local packing within the protein environment. (**C**) Conceptual depiction of the p.Met138Lys substitution, in which lysine introduces a positively charged terminal ammonium group that may plausibly influence local physicochemical properties or nearby intermolecular interactions. This schematic is conceptual and intended solely for illustrative purposes. It does not represent structural modeling, biophysical simulation, or experimental validation of pathogenicity.

This analysis identified a homozygous missense variant in *MYL2*, c.413T>A (p.Met138Lys). The variant affects a highly conserved methionine residue at codon 138 within the ventricular regulatory myosin light chain and is absent or extremely rare in population databases (gnomAD allele frequency < 0.00001, with no homozygotes reported). Multiple computational tools supported a deleterious effect, including SIFT (deleterious), PolyPhen-2 (probably damaging), MutationTaster (disease-causing), and CADD (scaled C-score > 20). In addition, ensemble and machine learning-based predictors demonstrated concordant results, with REVEL indicating a high pathogenicity score (>0.7) and AlphaMissense predicting a likely pathogenic effect, further supporting a deleterious functional impact. No additional pathogenic or likely pathogenic variants were detected that could plausibly account for the phenotype. Segregation analysis confirmed that both parents were heterozygous carriers, consistent with autosomal-recessive inheritance (Figure 2 and Figure 3). Methionine at residue 138 is conserved across representative orthologues, supporting functional constraint at the affected position, and the variant lies within a functionally relevant C-terminal region of the protein (Figure 2). Taken together, the rarity, segregation pattern, conservation profile, computational evidence, and phenotype consistency supported a likely pathogenic interpretation under ACMG/AMP criteria (Figure 3). A conceptual structural schematic further suggests that replacement of a hydrophobic methionine residue by a positively charged lysine could alter local physicochemical properties and nearby intermolecular interactions; however, this interpretation remains hypothesis-generating and does not constitute formal structural or functional proof (Figure 4).

While genetic testing was in progress, there was no sustained improvement in cardiac output or end-organ perfusion despite escalation of ventilatory, inotropic, and diuretic support. Once the molecular findings became available, they provided a unifying explanation for the child’s longstanding hypotonia, delayed motor development, and rapidly progressive cardiomyopathy. In light of the severity of myocardial dysfunction, established multiorgan failure, and the absence of realistic options for recovery or transplantation, the multidisciplinary team and family redirected care toward comfort and quality of life. Despite intensive supportive management, the clinical condition progressively worsened. Life-sustaining measures were gradually de-escalated with adequate analgesia and sedation, and the child died peacefully in the pediatric intensive care unit at 12 months of age, surrounded by his parents (Figure 1). No postmortem examination was performed.

The molecular diagnosis had immediate implications for both clinical care and family counseling. It excluded several previously considered metabolic, neuromuscular, and inflammatory explanations and instead supported a primary sarcomeric cardiomyopathy. Moreover, it clarified prognosis, informed goals-of-care discussions, and established a 25% recurrence risk for future pregnancies, thereby enabling counseling regarding carrier testing, prenatal diagnosis, and preimplantation genetic testing. This case also expands the allelic and phenotypic spectrum of recessive *MYL2*-associated cardiomyopathy and highlights the value of early genomic testing in infants with unexplained hypotonia accompanied by progressive cardiac disease.

## 3. Discussion

This case describes a lethal infantile cardiomyopathy associated with a homozygous *MYL2* c.413T>A (p.Met138Lys) variant and further extends the emerging spectrum of recessive *MYL2*-related disease. Most *MYL2* variants reported to date have been associated with autosomal dominant hypertrophic cardiomyopathy, often through altered myofilament calcium sensitivity and disturbed sarcomeric regulation [21,23]. By contrast, a smaller but increasingly important body of literature indicates that biallelic *MYL2* defects may produce a far more severe and early-onset phenotype. A homozygous splice-site variant was first reported to be associated with markedly reduced *MYL2* expression, type I fibre atrophy, and rapidly progressive neonatal cardioskeletal myopathy [24]. Subsequently, the same splice defect was further characterized, demonstrating impaired protein stability together with abnormal sarcomeric incorporation [25]. Likewise, a homozygous frameshift variant was identified in association with severe infantile cardiomyopathy and early death [26], whereas recessive *MYL2*-associated congenital fibre-type disproportion with cardiomyopathy has also been described [27]. The clinical course in the present child, namely congenital hypotonia followed by rapidly progressive systolic failure and multiorgan decompensation, is therefore consistent with the severe recessive end of the *MYL2* disease spectrum (Table 1).

At the same time, the absence of overt skeletal myopathy in our patient may indicate broader phenotypic heterogeneity, potentially related to the specific structural and functional consequence of the underlying allele. Importantly, emerging evidence suggests that the clinical expression of recessive *MYL2*-related disease may vary according to both variant class and protein location. Most reported cases of severe neonatal or infantile cardiomyopathy have involved biallelic truncating or splice-site variants, consistent with a loss-of-function mechanism associated with markedly reduced *MYL2* expression or defective sarcomeric incorporation [24,25,26,30]. In contrast, biallelic missense variants have also been reported in association with hypertrophic cardiomyopathy, particularly when they affect the N-terminal region of the protein [31,32]. Recent work has further emphasized the complexity of genotype–phenotype correlations in inherited cardiomyopathies and the importance of considering the structural and functional context of individual variants [33]. In this setting, the present case is notable because it involves a homozygous missense variant associated with a severe early-onset phenotype more typically seen with truncating alleles. This observation raises the possibility that certain missense substitutions affecting highly conserved residues within functionally important regions may have consequences comparable to those of loss-of-function variants, thereby further broadening the recognized phenotypic spectrum of recessive *MYL2*-associated disease.

**Table 1:** Comparative summary of reported biallelic *MYL2* variants and their associated clinical phenotypes in relation to the present case. Prior reports support a recessive *MYL2* disease spectrum ranging from severe neonatal or infantile cardiomyopathy, often with associated skeletal muscle involvement, to hypertrophic cardiomyopathy associated with biallelic missense variants. Most severe early presentations have involved splice-site or truncating alleles [24,25,26], whereas biallelic missense variants have been reported in both cardiomyopathic and hypertrophic phenotypes [27,34,35]. The present case extends this spectrum by showing that a homozygous missense variant in the C-terminal region may also be associated with a lethal early-onset phenotype.

Study	Variant	Variant Class	Approximate Protein Region	Age at Presentation	Cardiac Phenotype	Skeletal Muscle Involvement	Functional/Mechanistic Evidence	Clinical Outcome
Weterman et al. [24]	Homozygous splice-site variant	Splice-site/presumed loss-of-function	Not applicable	Neonatal	Cardiomyopathy	Present (type I muscle fibre disease)	Reduced *MYL2* expression reported	Early cardiac death
Zhou et al. [25]	Same homozygous splice-site variant as in Weterman et al. [24]	Splice-site/loss-of-function	Not applicable	Neonatal	Cardioskeletal myopathy with cardiomyopathy	Present	Impaired protein stability and abnormal sarcomeric incorporation	Early cardiac death
Manivannan et al. [26]	Homozygous C-terminal frameshift variant	Truncating/loss-of-function	C-terminal region	Infancy	Severe infantile cardiomyopathy	Not clearly reported	Molecular differences between dominant and recessive *MYL2* disease; consistent with severe recessive phenotype	Early death
Marttila et al. [27]	Homozygous missense variant associated with congenital fibre-type disproportion	Missense	Not specified	Infancy/childhood	Cardiomyopathy	Present (congenital fibre-type disproportion)	Suggested impaired sarcomeric regulation	Severe disease; variable outcome
Garcia-Pavia et al. [34]	Biallelic missense *MYL2*-associated genotype reported in end-stage hypertrophic cardiomyopathy cohort	Missense	Not clearly specified	Later onset/not neonatal	Hypertrophic cardiomyopathy	Not reported	Supports association of biallelic missense *MYL2* variation with hypertrophic phenotype	End-stage hypertrophic cardiomyopathy cohort
Allouba et al. [35]	Biallelic missense *MYL2*-associated genotype within hypertrophic cardiomyopathy genetic architecture study	Missense	N-terminal region reported in reviewer-cited HCM context/verify exact variant details from source before final lock	Later onset	Hypertrophic cardiomyopathy	Not reported	Supports recessive/biallelic missense contribution to hypertrophic cardiomyopathy in selected populations	Hypertrophic cardiomyopathy cohort
Present case	Homozygous *MYL2* c.413T>A (p.Met138Lys)	Missense	C-terminal region	Infancy	Severe dilated cardiomyopathy with marked left ventricular systolic dysfunction	No overt skeletal myopathy clinically recognized	Ultra-rare variant affecting a highly conserved residue; segregation consistent with autosomal recessive inheritance; deleterious in silico prediction; likely pathogenic interpretation under ACMG/AMP principles	Death at 12 months

The biological context of the p.Met138Lys substitution also supports the plausibility of disease association, although the mechanism should be interpreted cautiously. The affected residue lies within the C-terminal region of *MYL2*, in proximity to regions relevant to EF-hand-like structural organization, myosin-neck interaction, and regulation of cross-bridge kinetics [14,15]. Experimental studies have shown that regulatory light-chain variants can alter calcium binding, phosphorylation behavior, and strain-dependent ADP release, thereby modifying force generation and sarcomeric performance [20,21,22]. Moreover, ventricular regulatory light-chain phosphorylation is important for normal contractile function and myosin-head positioning, and disruption of this pathway has been linked to systolic dysfunction and ventricular remodeling in experimental systems [16,17,18,19]. Within that framework, replacement of a hydrophobic methionine residue by a positively charged lysine at a highly conserved site could plausibly disturb local physicochemical properties or nearby intermolecular interactions. Nevertheless, these considerations remain inferential. The present report does not provide direct structural or functional confirmation that p.Met138Lys alters myosin-binding geometry or contractile behavior, and the mechanistic interpretation should therefore be regarded as hypothesis-generating rather than demonstrative [20,23].

From a clinical perspective, this case also illustrates the diagnostic value of early genomic testing in infants presenting with hypotonia and otherwise unexplained heart failure. Such patients frequently undergo prolonged metabolic, neuromuscular, and imaging investigations before a unifying diagnosis is established [3,4]. However, exome- and genome-based approaches are increasingly showing meaningful diagnostic yields in critically ill infants with cardiomyopathy or severe cardiac failure and may identify causes not captured by phenotype-limited cardiomyopathy panels [5,6,7,9]. In parallel, rapid sequencing studies in neonatal and pediatric intensive care settings have demonstrated that genomic diagnoses can alter management, refine prognostic assessment, and inform decisions regarding escalation of care or palliation [10,11,12,13]. In the present child, conventional investigations were unrevealing, whereas trio-based exome sequencing provided a unifying molecular explanation for the combination of congenital hypotonia, delayed motor development, and fulminant cardiomyopathy. Accordingly, the diagnosis informed goals-of-care discussions and clarified recurrence risk for the family.

Several limitations should be acknowledged. First, no functional assays specific to p.Met138Lys were performed; thus, the biochemical consequences of the variant remain inferred from protein context, physicochemical substitution, and analogy to previously studied *MYL2* defects [20,25]. Second, muscle biopsy was not obtained, precluding direct assessment of *MYL2* expression, sarcomeric organization, or fibre-type abnormalities. Third, segregation analysis was limited to the parents, although this is not unusual in a rare autosomal recessive presentation. In addition, the parents were reportedly non-consanguineous despite the presence of a homozygous ultra-rare variant; while this does not exclude pathogenicity, distant unrecognized relatedness, founder effect, or regional allele enrichment remains possible explanations. No postmortem examination was performed, which further limits phenotypic correlation at the tissue level. Nonetheless, the variant is ultra-rare, affects a highly conserved residue, segregates appropriately within the family, is supported by multiple computational tools, and occurs in a clinical context strongly compatible with severe recessive *MYL2*-associated cardiomyopathy. Taken together, these features support a likely pathogenic interpretation under current ACMG/AMP principles, while still recognizing the absence of direct functional validation [28,29]. Future investigation should focus on defining the functional consequences of the Met138Lys substitution more directly. Structural modeling, biochemical assays, and in vitro testing in reconstituted systems or induced pluripotent stem-cell-derived cardiomyocytes would be particularly informative. In vivo approaches using zebrafish or *Drosophila* models may also help clarify whether this allele primarily disrupts cardiac sarcomeric performance or has broader effects relevant to combined cardioskeletal disease, as such systems have proved useful for the study of sarcomeric-variant biology [34,36]. As additional recessive *MYL2* variants are reported, more refined genotype–phenotype correlations may emerge and may help distinguish alleles associated with isolated cardiomyopathy from those producing combined neuromuscular and cardiac phenotypes. Ultimately, this case adds to the evidence that early genomic sequencing should be considered promptly in infants with unexplained hypotonia accompanied by severe early cardiac disease, in keeping with evolving precision-medicine frameworks for inherited cardiomyopathies [35,37,38].

## 4. Conclusions

We report a lethal infantile cardiomyopathy associated with a homozygous *MYL2* c.413T>A (p.Met138Lys) variant in a child who initially presented with congenital hypotonia and delayed motor development. This case broadens the allelic and phenotypic spectrum of recessive *MYL2*-associated disease and further supports the recognition of biallelic *MYL2* defects as a cause of severe early-onset cardiomyopathy, with or without overt skeletal muscle involvement. Although the structural and functional consequences of p.Met138Lys remain to be established experimentally, the combined evidence from rarity, segregation, conservation, computational prediction, and phenotype consistency supports a likely pathogenic interpretation. Moreover, this report highlights the clinical value of early genomic testing in infants with unexplained hypotonia and progressive cardiac dysfunction, particularly when conventional metabolic and neuromuscular investigations are unrevealing. Prompt molecular diagnosis may, therefore, clarify the underlying disease basis, inform prognosis, guide family counseling, and support timely decision-making in severe pediatric cardiomyopathy.

## Data Availability

The data presented in this study are available within the article. Additional data are not publicly available due to patient privacy considerations.

## References

[1] Towbin J.A., Lowe A.M., Colan S.D., Sleeper L.A., Orav E.J., Clunie S., Messere J., Cox G.F., Lurie P.R., Hsu D. (2006). Incidence, causes, and outcomes of dilated cardiomyopathy in children. JAMA.

[2] Lipshultz S.E., Sleeper L.A., Towbin J.A., Lowe A.M., Orav E.J., Cox G.F., Lurie P.R., McCoy K.L., McDonald M.A., Messere J.E. (2003). The incidence of pediatric cardiomyopathy in two regions of the United States. N. Engl. J. Med..

[3] Ware S.M. (2017). Genetics of paediatric cardiomyopathies. Curr. Opin. Pediatr..

[4] Kindel S.J., Miller E.M., Gupta R., Cripe L.H., Hinton R.B., Spicer R.L., Towbin J.A., Ware S.M. (2012). Pediatric cardiomyopathy: Importance of genetic and metabolic evaluation. J. Card. Fail..

[5] Bagnall R.D., Singer E.S., Wacker J., Nowak N., Ingles J., King I., Macciocca I., Crowe J., Ronan A., Weintraub R.G. (2022). Genetic basis of childhood cardiomyopathy. Circ. Genom. Precis. Med..

[6] Reuter M.S., Chaturvedi R.R., Liston E., Manshaei R., Aul R.B., Bowdin S., Cohn I., Curtis M., Dhir P., Hayeems R.Z. (2020). The Cardiac Genome Clinic: Implementing genome sequencing in pediatric heart disease. Genet. Med..

[7] Ritter A., Bedoukian E., Berger J.H., Copenheaver D., Gray C., Krantz I., Izumi K., Juusola J., Leonard J., Lin K. (2020). Clinical utility of exome sequencing in infantile heart failure. Genet. Med..

[8] Pezzoli L., Pezzani L., Bonanomi E., Marrone C., Scatigno A., Cereda A., Bedeschi M.F., Selicorni A., Gasperini S., Bini P. (2022). Not only diagnostic yield: Whole-exome sequencing in infantile cardiomyopathies impacts on clinical and family management. J. Cardiovasc. Dev. Dis..

[9] Keisling J., Bedoukian E., Burstein D.S., Gaynor J.W., Gray C., Krantz I., Izumi K., Leonard J., Lin K.Y., Medne L. (2024). Diagnostic yield of exome sequencing in pediatric cardiomyopathy. J. Pediatr..

[10] Saunders C.J., Miller N.A., Soden S.E., Dinwiddie D.L., Noll A., Alnadi N.A., Andraws N., Patterson M.L., Krivohlavek L.A., Fellis J. (2012). Rapid whole-genome sequencing for genetic disease diagnosis in neonatal intensive care units. Sci. Transl. Med..

[11] Dimmock D.P., Clark M.M., Gaughran M., Cakici J.A., Caylor S.A., Clarke C., Feddock M., Chowdhury S., Salz L., Cheung C. (2020). An RCT of rapid genomic sequencing among seriously ill infants results in high clinical utility, changes in management, and low perceived harm. Am. J. Hum. Genet..

[12] Kingsmore S.F., Cole F.S. (2022). The role of genome sequencing in neonatal intensive care units. Annu. Rev. Genom. Hum. Genet..

[13] Beaman M., Fisher K., McDonald M., Tan Q.K.G., Jackson D., Cocanougher B.T., Landstrom A.P., Hobbs C.A., Cotten M., Cohen J.L. (2022). Rapid whole-genome sequencing in critically ill neonates enables a precision medicine pipeline. J. Pers. Med..

[14] Han L., Mich-Basso J., Kühn B. (2021). Generation of human induced pluripotent stem cells and differentiation into cardiomyocytes. Methods Mol. Biol..

[15] Sheikh F., Lyon R.C., Chen J. (2015). Functions of myosin light chain-2 (*MYL2*) in cardiac development and disease. Gene.

[16] Janssen P.M.L. (2010). Kinetics of cardiac muscle contraction and relaxation are linked and determined by properties of the cardiac sarcomere. Am. J. Physiol. Heart Circ. Physiol..

[17] Dias F.A., Walker L.A., Arteaga G.M., Walker J.S., Vijayan K., Peña J.R., Ke Y., Fogaca R.T., Sanbe A., Robbins J. (2006). The effect of myosin regulatory light chain phosphorylation on the frequency-dependent regulation of cardiac function. J. Mol. Cell. Cardiol..

[18] Scruggs S.B., Hinken A.C., Thawornkaiwong A., Robbins J., Walker L.A., de Tombe P.P., Geenen D.L., Buttrick P.M., Solaro R.J. (2009). Ablation of ventricular myosin regulatory light chain phosphorylation in mice causes cardiac dysfunction in situ. J. Biol. Chem..

[19] van der Velden J., Papp Z., Boontje N.M., Zaremba R., de Jong J.W., Janssen P.M.L., Hasenfuß G., Stienen G.J.M. (2003). The effect of myosin light chain 2 dephosphorylation on Ca2+ sensitivity of force and cross-bridge kinetics in human failing hearts. Cardiovasc. Res..

[20] Greenberg M.J., Watt J.D., Jones M., Kazmierczak K., Szczesna-Cordary D., Moore J.R. (2010). Cardiomyopathy-linked myosin regulatory light chain mutations disrupt myosin strain-dependent biochemistry. Proc. Natl. Acad. Sci. USA.

[21] Szczesna D., Ghosh D., Li Q., Gomes A.V., Guzman G., Arana C., Zhi G., Stull J.T., Potter J.D. (2001). Familial hypertrophic cardiomyopathy mutations in the regulatory light chains of myosin affect their structure, Ca2+ binding, and phosphorylation. J. Biol. Chem..

[22] Kazmierczak K., Muthu P., Huang W., Jones M., Wang Y., Szczesna-Cordary D. (2012). Myosin regulatory light chain mutation found in hypertrophic cardiomyopathy patients increases isometric force production in transgenic mice. Biochem. J..

[23] Huang W., Szczesna-Cordary D. (2015). Molecular mechanisms of cardiomyopathy phenotypes associated with myosin light chain mutations. J. Muscle Res. Cell Motil..

[24] Weterman M.A., Barth P.G., van Spaendonck-Zwarts K.Y., Aronica E., Poll-The B.T., Brouwer O.F., van Tintelen J.P., Qahar Z., Bradley E.J., de Wissel M. (2013). Recessive *MYL2* mutations cause infantile type I muscle fibre disease and cardiomyopathy. Brain.

[25] Zhou Z., Huang W., Liang J., Szczesna-Cordary D. (2016). Molecular and functional effects of a splice-site mutation in the *MYL2* gene associated with cardioskeletal myopathy and early cardiac death in infants. Front. Physiol..

[26] Manivannan S.N., Darouich S., Masmoudi A., Gordon D., Zender G., Han Z., Fitzgerald-Butt S., White P., McBride K.L., Kharrat M. (2020). Novel frameshift variant in *MYL2* reveals molecular differences between dominant and recessive forms of hypertrophic cardiomyopathy. PLoS Genet..

[27] Marttila M., Win W., Al-Ghamdi F., Abdel-Hamid H.Z., Lacomis D., Beggs A.H. (2019). *MYL2*-associated congenital fiber-type disproportion and cardiomyopathy with variants in additional neuromuscular disease genes; the dilemma of panel testing. Cold Spring Harb. Mol. Case Stud..

[28] Richards S., Aziz N., Bale S., Bick D., Das S., Gastier-Foster J., Grody W.W., Hegde M., Lyon E., Spector E. (2015). Standards and guidelines for the interpretation of sequence variants: A joint consensus recommendation of the ACMG and the AMP. Genet. Med..

[29] Rehm H.L., Berg J.S., Brooks L.D., Bustamante C.D., Evans J.P., Landrum M.J., Ledbetter D.H., Maglott D.R., Martin C.L., Nussbaum R.L. (2015). ClinGen—The Clinical Genome Resource. N. Engl. J. Med..

[30] Ding Y., Bu H., Xu X. (2020). Modeling inherited cardiomyopathies in adult zebrafish for precision medicine. Front. Physiol..

[31] González-Rosa J.M., Burns C.E., Burns C.G. (2022). Zebrafish models of cardiac disease: From fortuitous mutants to precision medicine. Circ. Res..

[32] Fatkin D., Calkins H., Elliott P., James C.A., Peters S., Kovacic J.C. (2021). Contemporary and future approaches to precision medicine in inherited cardiomyopathies: JACC Focus Seminar 3/5. J. Am. Coll. Cardiol..

[33] Giugni F.R., Krieger J.E. (2023). Precision medicine in cardiomyopathies. ABC Heart Fail. Cardiomyop..

[34] Garcia-Pavia P., Vázquez M.E., Segovia J., Salas C., Avellana P., Gómez-Bueno M., Vilches C., Gallardo M.E., Garesse R., Molano J. (2011). Genetic basis of end-stage hypertrophic cardiomyopathy. Eur. J. Heart Fail..

[35] Allouba M., Walsh R., Afify A., Hosny M., Halawa S., Galal A., Fathy M., Theotokis P.I., Boraey A., Ellithy A. (2023). Ethnicity, consanguinity, and genetic architecture of hypertrophic cardiomyopathy. Eur. Heart J..

[36] Tamamitsu A.M., Nakagama Y., Domoto Y., Yoshida K., Ogawa S., Hirono K., Shindo T., Ogawa Y., Nakano K., Asakai H. (2021). Poor myocardial compaction in a patient with recessive *MYL2* myopathy. Int. Heart J..

[37] Kubo T., Morita H. (2021). The dawn of precision medicine in cardiomyopathies: Advance preparations of an ethnicity-specific database. Circ. J..

[38] Lipov A., Jurgens S.J., Mazzarotto F., Allouba M., Pirruccello J.P., Aguib Y., Gennarelli M., Yacoub M.H., Ellinor P.T., Bezzina C.R. (2023). Exploring the complex spectrum of dominance and recessiveness in genetic cardiomyopathies. Nat. Cardiovasc. Res..

